# SG-APSIC1092: Bundled preoperative preparation reduced surgical-site infections

**DOI:** 10.1017/ash.2023.92

**Published:** 2023-03-16

**Authors:** Nittaya Kaewtatip, Rossukon Kacharat

**Affiliations:** Naresuan University Hospital, Phitsanulok, Thailand; Naresuan University Hospital, Phitsanulok, Thailand

## Abstract

**Objectives:** We aimed to reduce the overall surgical-site infection (SSI) rate to 0.2%. **Methods:** A new checklist protocol was developed based on the APSIC guidelines. The bundle for preoperative preparation was implemented: adequate preoperative bathing, proper time of hand-forearm washing, and sufficient contact time of antiseptic application. The compliance rate was monitored with a weekly control chart from December 2019 to November 2020. **Results:** In total, 9,995 cases were operated at Narasuan University Hospital (NUH) in 2020, classified by surgical wound type as follows: clean wound, 62.6%; clean-contaminated wound, 32.1%; contaminated wound, 0.8%; and dirty wound, 4.5%. According to surgical wound type, the mean compliance with preoperative bathing was 68.22% for clean wounds, 68.33% for clean-contaminated wounds, and 34.82% for contaminated wounds. Hand hygiene preparation compliance was higher for clean wound surgeries (mean, 94.01%) and clean-contaminated wound surgeries (mean, 95.05%) than for contaminated wound surgeries (mean, 88.30%). A high percentage was achieved by the 3 groups. The rate of skin antiseptic preparation compliance was higher in the clean wound group (mean, 89.05%) and the clean-contaminated wound group (mean, 90.70%) than the contaminated wound group (mean, 68.12%). The lower rate might be due to time constraints in contaminated wound operations. Only 0.18% of clean-wound operations had SSIs, and the clean-contaminated wound group had 0.19% SSIs, whereas no SSIs occurred in the contaminated and dirty wound groups. The overall SSI rate was 0.17%; thus, we achieved our goal. **Conclusions:** A bundle of preoperative infection-prevention preparations reduced the rate of SSI. Furthermore, the bundle had a highly tangible positive impact for both internal and external stakeholders, and it was effective in ensuring good practice regarding preoperative preparation.

